# Artificial intelligence in pancreatic cancer

**DOI:** 10.7150/thno.77949

**Published:** 2022-10-03

**Authors:** Bowen Huang, Haoran Huang, Shuting Zhang, Dingyue Zhang, Qingya Shi, Jianzhou Liu, Junchao Guo

**Affiliations:** 1 Department of General Surgery, State Key Laboratory of Complex Severe and Rare Diseases, Peking Union Medical College Hospital, Chinese Academy of Medical Science and Peking Union Medical College, Beijing 100730, China.; 2 School of Medicine, Tsinghua University, Beijing, 100084, China.

**Keywords:** Artificial intelligence, machine learning, pancreatic cancer, early detection, prognosis prediction

## Abstract

Pancreatic cancer is the deadliest disease, with a five-year overall survival rate of just 11%. The pancreatic cancer patients diagnosed with early screening have a median overall survival of nearly ten years, compared with 1.5 years for those not diagnosed with early screening. Therefore, early diagnosis and early treatment of pancreatic cancer are particularly critical. However, as a rare disease, the general screening cost of pancreatic cancer is high, the accuracy of existing tumor markers is not enough, and the efficacy of treatment methods is not exact. In terms of early diagnosis, artificial intelligence technology can quickly locate high-risk groups through medical images, pathological examination, biomarkers, and other aspects, then screening pancreatic cancer lesions early. At the same time, the artificial intelligence algorithm can also be used to predict the survival time, recurrence risk, metastasis, and therapy response which could affect the prognosis. In addition, artificial intelligence is widely used in pancreatic cancer health records, estimating medical imaging parameters, developing computer-aided diagnosis systems, etc. Advances in AI applications for pancreatic cancer will require a concerted effort among clinicians, basic scientists, statisticians, and engineers. Although it has some limitations, it will play an essential role in overcoming pancreatic cancer in the foreseeable future due to its mighty computing power.

## Introduction

Pancreatic cancer (PC) is the deadliest form of all cancer. The five-year relative survival rate for PC is only 11% in the USA, which is the lowest among all cancers 1. There were 495773 new cases and 466003 deaths from PC worldwide in 2020, accounting for 2.6% of all new cancer diagnoses and 4.7% of all cancer deaths, respectively 2. In China, the incidence and mortality of PC among tumors are 2.47% and 3.64%, respectively 3. The main reason for such a poor prognosis of PC is the late diagnosis, with only about 20% of patients being diagnosed at an early stage. Most patients have non-specific first symptoms, such as jaundice, fatigue, change in bowel habits, and indigestion, that make it difficult to distinguish from non-cancer diseases 4. Most chemotherapy 5-7, targeted therapy 8, and immunotherapy 9-11 are ineffective because most patients are already in the progressive stage with local invasion and distant metastases at the detection time 12,13. A multicenter study demonstrated that patients with PC detected by screening had a 5-year survival rate of 73.3% and a median survival time of 9.8 years, compared with 1.5 years for patients with PC seen by non-screening 14. To diagnose early-stage PC accurately is desperately needed 15,16.

Radiographic imaging-based investigations 17-20 are fundamental techniques in PC screening, including endoscopic ultrasound (EUS), computed tomography (CT), magnetic resonance imaging (MRI), etc. Related techniques based on the above methods, such as EUS-guided fine needle aspiration (FNA) and biopsy (FNB), contrast-enhanced EUS (CE-EUS), CT (CE-CT), MRI (CE-MRI), and positron emission tomography-computed tomography (PET/CT), can further improve the accuracy of diagnosis. However, screening in the asymptomatic population is not recommended due to the economic burden and the relatively low incidence of PC in the general population 21,22. Also, early PC lacks biomarkers. Carbohydrate Antigen 19-9 (CA19-9), the best-validated biomarker in PC, does not have enough accuracy and specificity in screening early PC 23,24. Thus, many scientists are working to develop new early screening methods. Meanwhile, it is also essential to use better ways to assess treatment efficacy and prognosis, which will facilitate the development of appropriate clinical treatment options and critical drugs 25,26.

Artificial intelligence (AI) is a branch of computer science dedicated to producing a new kind of intelligent machine that can respond similarly to human intelligence 27. Nowadays, many researchers are attempting to apply AI to the medical field, including healthcare 28, oncology 29, cardiology 30, and more. Compared with traditional biometric methods, AI has greater flexibility and scalability, which allows it to be deployed for many tasks. Another advantage is its ability to integrate a large number of different data types and understand complex relationships between variables in a flexible, trainable manner. As the scale of medical data continues to expand and computer computing power continues to improve, AI is showing more and more advantages in processing big data. In clinical practice, AI can perform routine tasks consistently, freeing up physicians' time to solve more complex clinical problems 27,31. For PC, AI-assisted diagnostic techniques are also gaining more attention. The *2020 AI and Early Detection of Pancreatic Cancer Virtual Summit* discussed and highlighted the potential of AI in the early diagnosis of PC 32. In the recent meeting of *The Alliance of Pancreatic Cancer Consortia*, the discussion focused on imaging methods and the use of AI for the early detection of PC 33.

Despite the unparalleled advantages of AI, there are still many concerns about its application in the clinical field. For example, no one model can solve all problems, and all models have their range of adaptation 34. There is a risk that AI algorithms may ignore specific differences, such as gender and race, which can lead to bias because of the heterogeneity between the training set and other patients 35-37. AI also faces many ethical issues in clinical practice, such as the need for researchers and healthcare organizations to protect data from hacking for patient privacy. Healthcare systems should strive to ensure that the benefits of AI are passed on to all patients they serve, not just those with access to more resources. Also, it is difficult to assign liability for medical malpractice arising from defects in AI 37,38. In addition, AI's transparency and interpretability are challenged by factors such as patient privacy, algorithm interpretability, publication bias, etc. 39,40. Most of the AI devices approved by the FDA have only undergone retrospective studies. The lack of prospective studies may lead to unexpected conditions during the clinical application of AI devices 41,42. Solving these problems is a key point for the future of AI in clinical applications.

In this review, using “artificial intelligence”, “machine learning”, and “pancreatic cancer” as the keywords, we searched the relevant literature published by July 2022 in PubMed, Embase, Web of Science, and other databases. We summarized the application of AI in several aspects of PC. Compared with the existing studies, our review summarizes more comprehensively 43,44. We outlined how AI could help in medical image analysis, pathological examination, and biomarkers in the tumor diagnosis process. In prognosis respect, the AI analysis includes survival time, recurrence risk, metastasis, and therapy response. Finally, we summarized the current status of AI in PC and discussed the future challenges and directions for the field.

## State-of-the-art AI Algorithms involved in Pancreatic Cancer

### Machine Learning

**Figure 1:**
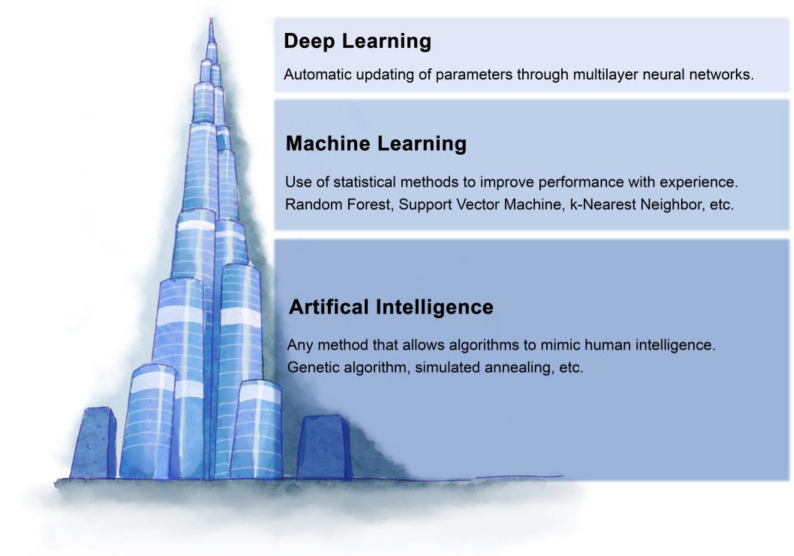
The relationship between artificial intelligence, machine learning, and deep learning. Artificial intelligence refers to the use of machines to simulate human intelligence. Machine learning is a subfield of artificial intelligence, which mainly studies how to simulate or realize the learning function in human intelligence. The deep learning model is a subset of machine learning, which is a model combining multi-layer neural networks.

**Figure 2:**
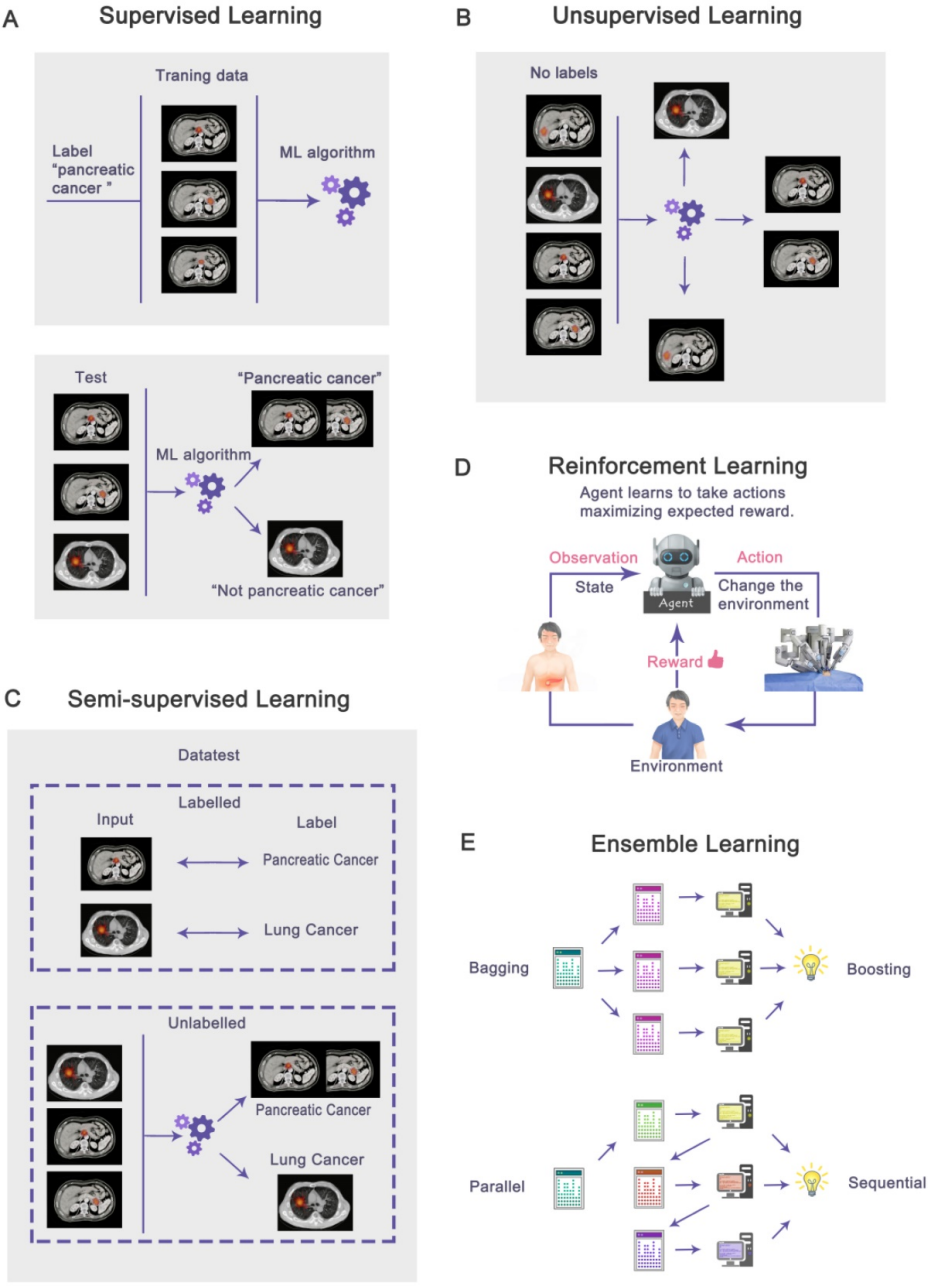
Based on labels, machine learning can be classified as supervised (**A**), unsupervised (**B**), semi-supervised (**C**), reinforcement learning (**D**), and ensemble learning (**E**) that integrates multiple algorithms. In supervised learning, all data is labeled, while unsupervised learning is unlabeled. Semi-supervised learning contains a small amount of labeled data and a large amount of unlabeled data. Reinforcement learning is when the agent interacts with the unknown environment and obtains rewards or punishments from the environment. In ensemble learning, multiple algorithms are integrated to solve problems. The algorithms may be parallel (Bagging) or sequential (Boosting).

Machine learning (ML) is a subfield of AI that solves the problem of how to build computers that improve automatically through experience (**Figure 1**). Based on a large amount of feature data, ML can use specific algorithms to learn how to accomplish a task 45. In today's medical field, there is a massive amount of data generated every day, and it becomes a challenge to integrate this data to make predictions. The most significant advantage of ML is the ability to integrate vast amounts of data and combine the observed and predicted quantities in nonlinear and highly interactive ways 46. ML techniques can be broadly classified based on the type of labels. Based on labels, machine learning can be classified as supervised, unsupervised, semi-supervised, and reinforcement learning. There is also ensemble learning that integrates multiple algorithms (**Figure 2A-2E**).

Receiver operating characteristics (ROC) curves help organize ML classifiers and visualize their performance. ROC curve is a line graph plotted with sensitivity as the vertical coordinate and (1-specificity) as the horizontal coordinate. The area under the ROC curve (AUC) is the evaluation metric, and the larger the AUC value, the better the corresponding algorithm performs 47. Other metrics, including accuracy, sensitivity, specificity, F1-Score, positive predictive value (PPV), and negative predictive value (NPV), are also commonly used to evaluate the result of the ML 48.

#### Supervised Learning

Supervised learning is constructing a model in which each observation vector has a corresponding response variable. In other words, all data is labeled. By fitting a model that relates responses to predictors, supervised learning can accurately predict future observed responses or better understand the relationship between responses and predictors 49,50. Examples of such algorithms include Logistic Regressions (LR), Decision Trees (DT), Support Vector Machines (SVM), Naïve Bayes (NB), Artificial Neural Networks (ANN), etc., and the best application scenario for each algorithm varies 51. In this review, most of the algorithms used in PC are supervised learning. A typical application of supervised learning algorithms is the precise diagnosis, including detection, grading, and differential diagnosis, using radiomics, digital pathology slides, or biomarkers. The prognosis of PC is also widely used to predict survival time, recurrence rate, metastasis, and therapy response.

#### Unsupervised Learning

Unsupervised learning means we can know the observation vector, not the associated response. In other words, all data is unlabeled. Using the observation vector's data makes it possible to perform clustering, correlation evaluation, dimensionality reduction, etc. 50,52. Examples of such algorithms include K-mean clustering 53, Principal Component Analysis (PCA) 54, Non-negative Matrix Factorization (NMF) 55, etc. The application of unsupervised learning in PC is relatively rare, but there have been attempts to do so, including classification 56, feature extraction of CT images 57,58 or pathological slides 59, and estimation of medical imaging parameters 60.

#### Semi-supervised Learning

As its name suggests, semi-supervised learning is somewhere between supervised and unsupervised learning, allowing the use of large amounts of available unlabeled data in combination with small labeled datasets in many use cases. Semi-supervised learning can utilize a small amount of labeled data to obtain better performance than supervised learning while utilizing less labeled data to achieve the same level of performance close to that of supervised learning 61,62. Examples of such algorithms include generative models, self-training, co-training, graph-based learning, Semi-supervised support vector machines, etc. 61,63.

The application of semi-supervised learning is mostly seen in medical imaging. Supervised learning algorithms may lack annotated data because annotation of medical imaging data is time-consuming and requires a high level of expertise. By using semi-supervised learning algorithms, the task of segmentation or diagnosis using medical images can be accomplished with fewer annotations 64. For example, CT images of PC can be used for segmentation and diagnosis 65.

#### Reinforcement Learning

The reinforcement learning process is guided by a specific goal. Agents interact with the unknown environment and get reward or punishment feedback from the environment. Then, it uses this feedback to train itself and collect experience and knowledge about the environment to achieve specific goals 66. Reinforcement learning can also be combined with deep learning to become deep reinforcement learning. It uses dynamic data and labels to bring feedback signals into the learning process rather than constructed, static dataset labels as in traditional machine learning 67.

Reinforcement learning algorithms are commonly used in decision-making in the medical field. Due to the heterogeneity of patients' conditions and treatment responses, it is challenging to realize precision medicine. Reinforcement learning can construct dynamic treatment regimens that consider the immediate effect of treatment and the long-term benefit to the patient 68,69. For PC, reinforcement learning algorithms can generate high-quality treatment plans for pancreas stereotactic body radiation therapy (SBRT) to achieve optimal metering distribution 70.

#### Ensemble Learning

Rather than a single algorithm, ensemble learning seamlessly integrates various machine learning algorithms into a unified framework, typically for supervised learning. Specifically, ensemble learning samples the data and produces prediction results using multiple learners. The above results are combined, and the errors of individual learners are potentially compensated by other learners, resulting in better prediction performance 71,72. Depending on whether the different learners are independent of each other, the ensemble approach can be divided into two main frameworks: the dependent and independent 71. The output of each learner of the dependent framework affects the next learner, which is represented by AdaBoost in the “Boosting” algorithm 73. In an independent framework, individual learners can output in parallel, which is represented by Random Forest (RF) in the “Bagging” algorithm 74. Both dependent and independent frameworks have applications in diagnosing 75-77 and prognosis 78-81 PC.

### Deep Learning

**Figure 3:**
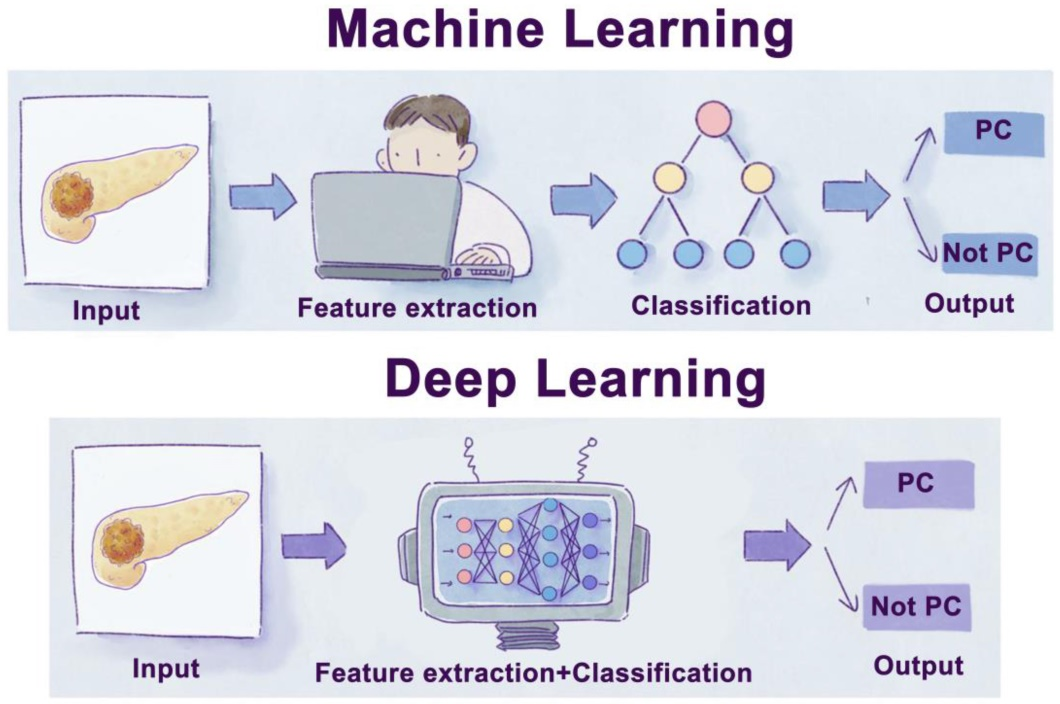
Schematic diagram of machine learning and deep learning process. Traditional machine learning usually needs four steps: input, feature extraction, classification, and output. Moreover, deep learning is a subset of a machine learning algorithm, which can extract labels by itself without manual extraction.

Deep learning (DL) is a subset of ML algorithms (**Figure 1**). It allows a machine to feed raw data and automatically build complex concepts. Take image recognition as an example. The mapping from many different pixels to an image is very complex. DL solves this difficulty by decomposing the complex mappings required to recognize an image into a series of simple nested mappings. The algorithm can be divided into one visible layer and several hidden layers. The visible layer is where the image is fed, while hidden layers are where the algorithm gradually extracts the features from the image 82,83. Compared to shallow ML and traditional data analysis methods, DL models have superior performance in many applications, especially in domains with extensive and high-dimensional data. However, shallow ML performs better for low-dimensional data, especially when a limited training set 84. With the significant development of computer technology, many DL algorithms, such as Convolutional Neural Networks (CNN), Recurrent Neural Networks (RNN), MultiLayer Perceptron (MLP), Generative Adversarial Networks (GAN), and Deep Belief Networks (DBN), have been widely used in the field of oncology (**Figure 3**) 85-87.

## AI in Tumor Diagnosis Process

Reading medical images to make judgments is essentially a problem of recognizing complex patterns, which computers can be trained using ML models to achieve efficient and repeatable recognition. AI can play a role in several steps in medical image-based PC diagnosis, including image reconstruction, segmentation, and detection, characterization, grading of pancreatic disease based on image features. Using similar techniques, AI can also identify digitized histopathology slides. It can potentially improve the accuracy, reproducibility, and efficiency of diagnosis using histological sections. In addition, the computer can analyze biomarker information with high throughput and accuracy, thus identifying tumor-related biomarkers more efficiently and using this information for diagnosis.

### AI in Medical images-based diagnosis

Imaging techniques play an essential role in the diagnosis of PC. Current clinical imaging modalities include EUS, CT, MRI, and PET, with different advantages and disadvantages in clinical applications (**Table 1**). In the traditional process of medical image analysis, experienced radiologists are required. With AI technology, it is possible to free imaging physicians from tedious and repetitive labor to handle tasks that require more creativity.

Radiomics refers to the high-throughput extraction of many image features from radiographic images, which may be challenging to recognize or quantify by the human eye. Radiomics can be used to identify lesions, allowing for early detection and diagnosis of disease. Also, radiological features can predict prognoses, such as survival, tumor metastasis, and treatment response, and correlate with genomic, transcriptomic, or proteomic features 88-91. Conventional workflow in radiomics usually contains four steps: image acquisition, segmentation, feature extraction, and analysis 92. For image acquisition, standard protocols are needed to minimize confounding variables 93. Segmentation involves identifying the images' regions of interest (ROIs) and defining the boundaries in the pictures. While this step can be done manually by practiced radiologists, many ML methods have been used for image segmentation 88,94,95. The dice similarity coefficient (DSC), used to measure the similarity of two sets, is the most used metric in evaluating segmentation performances. In some research, segmentation performances also used Hausdorff distance and intersection over union for evaluation 96. The next step is extracting radiomics features from ROIs, including histogram-based, texture-based, model-based, transform-based, and shape-based features. Radiomic features are usually numerous, highly correlated, and redundant features that need to be filtered out before they can be used for model building 91,94. The final step is to build a predictive model using ML and evaluate the model's performance.

#### Endoscopic Ultrasound

EUS is widely used in diagnosing pancreatic lesions because it provides high-resolution images of the pancreas without being disrupted by gas, bone, or subcutaneous fat. EUS and its related techniques, such as CE-EUS and EUS elastography, show high specificity and sensitivity in diagnosing pancreatic diseases. Furthermore, it is frequently used to identify regional lymph nodes and assess the relationship of tumors to nearby vascular structures 19,97. In addition, EUS can guide tissue sampling to obtain pathological information about the cancerous tissue 20,98. The disadvantage is that EUS is an invasive procedure with a risk of pancreatitis or bleeding. The method is also demanding on the operator, and improper handling may reduce the accuracy of the diagnosis 99,100. As early as 2001, scientists had researched using neural networks to enhance EUS to detect and diagnose PC. Many studies have emerged in recent years (**Table 2**) 101-112.

AI in EUS is frequently used to aid in the differential diagnosis of PC and other conditions. In our statistics, a few studies applied AI in the differential diagnosis of PC. The overall AUC, accuracy, sensitivity, and specificity were 0.940-0.986, 80%-98.26%, 87.59%-100%, 50%-93.38%, respectively 101-109. Udriştoiu *et al.* combined CNN and long short-term memory (LSTM) neural networks to construct an ML algorithm for differential diagnosis of focal pancreatic masses, using multi-sequences EUS (grayscale, color Doppler, arterial and venous phase contrast-enhancement, and elastography). Their model achieved high AUC and accuracy among the studies 105. Kuwahara *et al.* used CNN (ResNet-50) in turn to extract image features and distinguish between benign and malignant intraductal papillary mucinous neoplasm (IPMN) 110.

The physician can also use AI in EUS for pancreas segmentation. Iwasa *et al.* analyzed 100 patients with different PC. Segmentation was performed using U-Net with 100 epochs and was evaluated with 4-fold cross-validation. The median intersection over the union of all cases was 0.77 111. Zhang *et al.* developed a station classification model and a pancreas segmentation model for EUS training and quality control. The DSC of the pancreas segmentation model was 71.5%, and the accuracy of the station recognition model reached 82.4% 112.

#### Computed Tomography

CT is the dominant imaging modality for diagnosing and staging PC, which is more widely available and less expensive than other imaging modalities. Due to the high spatial resolution of CT can be used for diagnosing and staging the tumor, identifying vascular involvement, tumor resectability analysis, etc. However, the tumor may not be visible due to the poor contrast resolution of CT. With the use of multiplanar reformations, 3D techniques, and spatial and temporal resolution improvement, CT has achieved high sensitivity (96%) in tumor identification 18,97. AI can assist CT-based diagnosis in many ways, including pancreas segmentation, diagnosis and staging of PC, differential diagnosis, and resectability analysis (summarized in **Table 3**).

In our statistics, several studies focused on PC or PC precursor lesions diagnosis or prediction by AI-assisted CT. Their AUC, accuracy, sensitivity, specificity were 0.79-0.999, 77.66%-99.2%, 76.64%-100%, 85.59%-98.5%, respectively 113-122. The method of Chu *et al.* 120 has the highest accuracy (99.2%) among the studies. 190 pancreatic ductal adenocarcinoma (PDAC) patients and 190 healthy control cases with 64-MDCT scans were included, and 0.75-mm slices of venous phase images were chosen for segmentation and radiomics analysis. Images were manually segmented, and their features were extracted by a binary mask and selected by minimum-redundancy maximum-relevancy feature selection. Finally, an RF classifier was constructed to classify PDAC from the normal pancreas. All of the PDAC cases were correctly classified. Only one normal case from a renal donor was classified as PDAC, giving an AUC of 0.999, an accuracy of 99.2%, a sensitivity of 100% and a specificity of 98.5%.

Some studies focused on the differential diagnosis of pancreatic disease. Ikeda* et al.* investigated a neural network classifier for the differential diagnosis of PDAC and mass-forming pancreatitis, with an AUC of 0.866 123. Chen *et al.* combined imaging features and enhanced CT texture analysis. Then they used LASSO and RFE_LinearSVC algorithms to select features to differentiate pancreatic serous cystadenomas (SCN) from pancreatic mucinous cystadenomas (MCN), with an AUC of 0.932 124. Ren *et al.* extracted 792 radiomics features from the late arterial and portal venous phases of CE-CT. They then used an RF classifier for differential diagnosis between pancreatic adenosquamous carcinoma (PASC) and PDAC and achieved an AUC of 0.98 125. Xie *et al.* extracted and screened ten optimal imaging features and applied an RF algorithm to build a Rad-score to discriminate between MCN and atypical SCN. The method achieved an AUC of 0.97 126. Li *et al.* extracted 1409 radiomics features from the portal phase of multidetector computed tomography (MDCT). After removing irrelevant features and Bonferroni correction, four features by LASSO regression were still significantly associated with focal-type autoimmune pancreatitis (AIP) and PDAC. The LASSO logistic regression formula was used to obtain the rad-score for discriminating focal-type AIP from PDAC (AUC 0.97) 127. In addition to using traditional algorithms (PyRadiomics), Ziegelmayer *et al.* used deep CNN for radiomics feature extraction. For the prediction of AIP or PDAC, an extremely randomized tree classifier was fit on the extracted features with an AUC of 0.90 128. Yang *et al.* adopted the RF method to construct a diagnostic prediction model based on textural parameters of CE-CT images to discriminate between SCN and MCN 129.

Several studies used AI-assisted CT for pancreas or PC segmentation. Their DSCs ranged from 60.6% to 91% 65,130-135. Panda *et al.* developed a two-stage 3D CNN model based on a modified U-net architecture. 1917 portal venous phase CT scans with normal pancreas were used for training, validation, and testing. The mean DSC of their method was 91%, the highest among the studies. The authors also demonstrated that their approach could be applied to CT images containing PC (mean DSC=0.96) 130. Zhou *et al.* developed a 4DCT-based method for tumor positioning without pancreas segmentation. Using 4DCT, they built a digitally reconstructed radiograph dataset for each patient, with clinical target volume (CTV) contours labeled. Then the datasets trained the ResNet and FPN algorithm to predict CTV. DSC of their method was 98%, which shows the accuracy of the positioning 136.

For other applications of AI in CT, Abel *et al.* developed and evaluated an algorithm based on a two-step nnU-Net architecture for automated detection of pancreatic cystic lesions (PCL) in CT 137. Lyu *et al.* used high strength levels of the DL image reconstruction (DLIR-H) algorithm to predict the resectability of PC 138. Chang *et al.* extracted radiomics features of CE-CT images by the SVM model and generated a radiomics signature by the LASSO model for the preoperative prediction of histological grades of PDAC. The radiomics signature for the training set and external validation data had an AUC of 0.961 and 0.770, respectively 139. Luo *et al.* built a CNN-based model to analyze CE-CT images for pancreatic neuroendocrine neoplasms (PNET) grading 140. Wan *et al.* built handcrafted, SAE, and hybrid features-based SVM prediction models for PNET grading. Among them, the hybrid feature model performs best (AUC 0.771) 57.

In addition to using CT image features to aid the precise diagnosis of PC, some studies attempted to improve CT image quality. Ohira *et al.* constructed a deep CNN that generates virtual monochromatic images from single-energy computed tomography (SECT) images for improved PC imaging quality 141. Noda *et al.* used a DL image reconstruction algorithm to reconstruct dual-energy computed tomography images, thus assisting PC diagnosis 142.

#### Magnetic Resonance Imaging

MRI is of great value in diagnosing PC due to its ability to collect many types and superior soft-tissue contrast. The best application circumstances for MRI include: (1) detection of small non-contour-deforming tumors, (2) evaluation of local extension and vascular encasement, and (3) determination of lymph node, liver, and peritoneal metastases 17,144. However, MRI is usually more expensive than CT. Also, metal implants may cause image artifacts and hinder imaging 145. The applications of AI in MRI-based diagnosis include pancreas segmentation, PC classification, and grading (summarized in **Table 4**) 56,146-152.

Li *et al.* collected four modalities of MRI for PC segmentation. Since MRI image labeling is time-consuming and laborious, they attempted to train the algorithm on labeled MRI images in one modality and test the model's performance in another modality to achieve unsupervised labeling of MRI images. The DSC of their methods on different models are 62.08% (T1), 61.35% (T2), 61.88% (DWI), and 60.43% (AP) 146. To achieve pancreas segmentation, Chen *et al.* developed a spiral-transformation algorithm to map 3D images onto a 2D plane. Combined with U-Net, their method has a relatively high mean DSC (65.60%) 147. Liang *et al.* trained the CNN with Stochastic Gradient Descent with Momentum algorithm, and their method got 71% DSC 148.

Four teams used AI-assisted MRI to classify or grade PC. Goldenberg *et al.* built three groups of tumor models with different types of PDAC cells. The method of support vector machine predicted the correct tumor model with 87.5% (CEST MRI) and 85.1% accuracy (DCE MRI) 149. Cui *et al.* used multivariate logistic regression to analyze the extracted MRI image features, and the AUC of their method was 0.903 150. Corral *et al.* used a CNN to classify IPMN. Sensitivity and specificity to identify high-grade dysplasia or cancer were 75% and 78%, respectively. Moreover, the AUC was 0.78 151. Hussein *et al.* were among the few teams using an unsupervised learning algorithm. 3D CNN was used to classify IPMN. Their method's accuracy, sensitivity, and specificity were 58.04%, 58.61%, and 41.67% 56.

Cheng *et al.* compared the predictive value of two medical imaging methods for predicting malignant IPMN. Radiomics features were extracted from arterial and venous phase images of CT and T2-weighted images of MRI, respectively. The LASSO algorithm was used for feature selection, and LR and SVM algorithms were applied to construct radiomics models. The results show that the MRI-based model (AUC 0.940) has a better performance compared to the CT-based model (AUC 0.864) 152.

#### Positron Emission Tomography

Positron Emission Tomography (PET) is a molecular imaging technique that has a vital role in diagnosing and staging tumors (**Table 5**). In combination with CT technology, PET/CT can help localize functional abnormalities and provide information on the biological characteristics of the tumors, such as metabolism, hypoxia, and proliferation 153,154. Fluorine 18-fluorodeoxyglucose (FDG), a glucose analogue, is the most widely used radiotracer in PET. However, glucose metabolism is not specific, and physiological uptake of FDG by inflamed tissues may lead to false-positive results 155. Also, FDG intake is reduced in patients with hyperglycemia, leading to a false negative result 156. For PC, FDG-PET is more sensitive than CT for treatment monitoring after radiotherapy and description of recurrence after tumor resection 17.

Li *et al.* used simple linear iterative clustering on PET/CT pseudo-color images for pancreas segmentation and developed threshold component analysis to select the most beneficial feature combination. Then they designed a hybrid feedback-support vector machine-random forest (HFB-SVM-RF) model to identify normal pancreas or PC. The DSC and Jaccard index for pancreas segmentation is 78.9% and 65.4%, respectively. For PC diagnosis, the accuracy and sensitivity of their method were 96.47% and 97,51%, respectively 75. Liu *et al.* extracted 502 radiomics features from dual-time PET/CT and used SVM to build a classifier to distinguish between PDAC and AIP. The AUC, Accuracy, Sensitivity, and Specificity were 0.9668, 89.91%, 85.31%, and 96.04%, respectively 157. Xing extracted 251 expert-designed features from PET/CT images and combined them into five feature sets according to their modalities and dimensions. Four feature selection strategies (Spearman's rank correlation coefficient, minimum redundancy maximum relevance, support vector machine recursive feature elimination, and no feature selection) and four machine learning classifiers (RF, adaptive boosting, SVM with the Gaussian radial basis function, and SVM with the linear kernel function) were used to found the optimal feature set. Based on the best combination of the feature selection strategy and classifier, the model that differentiates AIP from PDAC was developed and achieve an AUC of 0.93 158. Xing *et al.* extracted radiomics features from PET/CT images using Pyradiomics and used the XGBoost algorithm to build a prediction model for PDAC pathological grade prediction 159.

### AI-assisted Pathological Examination

In addition to the above radiographic images, The pathologist can apply AI to Hematoxylin and Eosin (H&E)-stained or immunofluorescent-stained whole slides images (WSI) for PC diagnosis 160. FNA and FNB are essential diagnostic methods for suspended PC with high accuracy 161,162. AI can assist in the diagnosis of PC by analyzing cytology and biochemical characteristics of FNA/FNB samples. Here, we summarized the application of AI in pathological examination (**Table 6**) 76,163-169.

Song *et al.* constructed a system to automatically segment epithelial cell nuclei on slide images and extract morphological features. They subsequently used multiple classifiers to demonstrate the effectiveness of the designed method in the differential diagnostic of SCN and MCN 163. Using a similar approach, Song also worked on diagnosing and grading PDAC 164. Kriegsmann *et al.* used CNN to construct models for automatic localization and quantification of tissue categories in whole tissue slides, including pancreatic intraepithelial neoplasia and PDAC 165. Ki67 index has clear guidance for proliferation rate and grading of PNET. However, the non-specificity of Ki67 staining and counterstaining hinders the accurate quantification of the Ki67 index. Therefore, Niazi *et al.* proposed a DL method based on Ki67-stained biopsy images to distinguish NET from non-tumor areas automatically and achieved 97.8% sensitivity and 88.8% specificity 166. Vance *et al.* combined WSI and cyclic multiplexed immunofluorescence to collect 31 markers of PDAC and used an RF algorithm to identify tumor cell populations, achieving an accuracy of 87% 76.

Momeni-Boroujeni *et al.* used a K-means clustering algorithm to segment cell clusters from FNA-based slides, extracted the morphological features of the cell clusters, and trained a multilayer perceptron neural network (MNN) with these features. Then tested their ability to discriminate between benign and malignant pancreatic cytology (accuracy 100%) 167. Vance *et al.* trained the CNN using FNB-based slides to assess PDAC and achieved an AUC of 0.984 168. Kurita *et al.* combined biomarkers in the cyst fluid, cytological features obtained by FNA, and clinical variables to training neural networks for differentiating malignant and benign PCLs 169.

### The applications of AI in Biomarkers

Biomarkers have a significant role in diagnosing, staging, and treating PC. The use of appropriate biomarkers for screening in high-risk populations is an essential aspect of the early diagnosis of PC. However, the current PC biomarkers lack sufficient sensitivity and specificity for clinical application 44,161,170. Liquid biopsies allow a comprehensive cancer profile to be evaluated in a non-invasive and real-time manner and help discover more data for cancer research, including CTCs, cfDNA, exosomes, etc. The biomarkers in these data can be analyzed using AI for their association with diseases. With the expansion of data obtained from liquid biopsies, scientists can better apply AI to biomarker-based early diagnosis of cancers 161,171,172.

As summarized in **Table 7**, various biomarkers have been used to diagnose or detect PC with the aid of AI, including exosomes 173-175, proteins 176-179, cell-free DNA (cfDNA) 77, circulating microRNA 180, extracellular vesicles long RNA 181, gene expression pattern 182, etc. The above studies mainly contain two categories, 1) biomarkers were known, these data trained ML algorithms to obtain prediction models for PC 173,178,179,181,182, and 2) biomarkers were uncertain, features in the dataset needed to be extracted, then ML models were used to evaluate the value of these features in the diagnosis of PC 77,174-177,180.

#### Genomics

All cancers occur due to a series of mutations in the cellular genome. The most common driver genes of PDAC are *KRAS, CDKN2A, TP53,* and* SMAD4*, and genetic alterations of the SWI/SNF and COMPASS complexes significantly impact PC. The genome can be used as biomarkers for PC diagnosis, and genome studies can also reveal features that make PC therapeutically susceptible 183.

A part of some researchers used AI to analyze the existing genomic data in the database to find their association with PC. Wang *et al.* used 78 PDAC samples from the GEO database as the training set. By combining Support Vector Machine Recursive Feature Elimination (SVM-RFE) and Large Margin Distribution Machine Recursive Feature Elimination (LDM-RFE) algorithms, they predicted seven differentially expressed genes as specific biomarkers for PC 184. Ko *et al.* developed a Gene Vector for Each Sample (GVES) model, which generated vector representations of genes using gene expression and biological network data from the TCGA database. In cases of small sample sizes, GVES had good accuracy for predicting prognostic genes 185. Cristiano *et al.* developed an approach to evaluate fragmentation patterns of cfDNA across the genome and constructed a gradient tree boosting (GBM) model to detect cancer. For PC, the AUC, accuracy, and specificity were 0.86, 67%, and 71%, respectively 77.

#### Transcriptomics

Both coding and non-coding RNAs are essential for gene expression. In addition to mRNAs directly related to protein expression, many non-coding RNAs are involved in or reveal tumor progressions, such as miRNA, lncRNA, and circRNA 186. The study of PC transcriptomics helps to understand the mechanism of tumor progression and provides valuable prognostic markers 187.

Xuan *et al.* obtained miRNA-disease association data from the human miRNA-disease database and built a network representation learning and CNN-based model to predict disease miRNAs. The AUC of the model in PC miRNA prediction is 0.971 188. Some researchers have also identified biomarkers directly from pathological samples. Mori *et al.* directly sequenced the RNA from PDAC tumor tissues and normal tissues, and DL analyzed the data. The selected genes were all important prognostic factors for PC based on the TCGA database 189. Alizadeh Savareh *et al.* identified a series of circulating miRNAs associated with PC by analyzing four GEO microarray datasets. The value of the top miRNAs was then assessed by ML methods (Particle Swarm Optimization (PSO) + ANN and Neighborhood Component Analysis (NCA)). The final model, which consist of five miRNAs (miR-663a, miR-1469, miR-92a-2-5p, miR-125b-1-3p and miR-532-5p), showed good diagnostic results on the investigated cases and validation set (Accuracy: 0.93, Sensitivity: 0.93, Specificity: 0.92) 180. Yu *et al.* analyzed the extracellular vesicles' long RNA profile of PDAC, chronic pancreatitis (CP), and healthy plasma samples. The d-signature was identified using an SVM algorithm to detect PDAC (0.960), identify resectable stage I/II cancer (AUC 0.949), and distinguish PDAC from CP (AUC 0.931) 181. Almeida *et al.* identify five differentially expressed genes (*AHNAK2, KRT19, LAMB3, LAMC2,* and* S100P*) from a gene expression microarray meta-analysis to train an ANN to classify PDAC or healthy samples. The ANN model could classify the test samples with a sensitivity of 87.6% and a specificity of 83.1% 182.

#### Proteomics

Proteins are the performers of gene-encoded functions. Although proteins are the direct products of mRNA translation, many unexpected associations in the proteome are primarily absent in RNA. Tumor proteome is closely related to epithelial and mesenchymal markers, cell lineage sensitivity, etc. Interventions on the proteome also provide new avenues for tumor treatment 190.

Gao et al. used SELDI-TOF-MS to analyze serum proteomes from PC patients and healthy controls. SVM analysis of the spectra was used to generate a predictive algorithm based on maximally differentially expressed proteins between PC patients and healthy controls. Four significant peaks were used to build a classifier to distinguish PC patients from healthy controls. Combining the SELDI protein peaks and CA19-9, their classifier achieves the AUC of 0.971 176. Yu *et al.* used SDLDI-Proteinchip to analyze serum protein profiling from PC patients and healthy controls. A decision-tree algorithm was trained to separate PC from controls. The sensitivity and specificity of their method were 88.9% and 74.1%, respectively 177. Yang *et al.* used three tumor markers (CA19-9, CA125, and CEA) from serum specimens to train the ANN model for PC diagnosis. The AUC, accuracy, sensitivity, and specificity were 0.905, 83.53%, 90.86%, 67.50%, respectively 178.

#### Exosomes

Exosomes are extracellular vesicles secreted by eukaryotic cells involved in intercellular communication containing nucleic acids, proteins, lipids, and glycoconjugates. It can regulate tumor cell proliferation, metastasis, Epithelial-to-Mesenchymal Transition (EMT), and angiogenesis during tumor development. In clinical practice, it can be used as a biomarker for tumor diagnosis, grading, and prognosis prediction 191,192. Many studies have used exosomes to diagnose, treat, and monitor treatment response in PC 193.

Chen* et al.* developed a quantitative analysis platform for continuously quantifying multiple exosomal surface biomarkers from blood samples. Four exosomal surface biomarkers (HER2, GPC-1, EpCAM, EGFR) were immunostained to calculate the number. Linear discriminant analysis was further used to identify exosomes from pancreatic and breast cancer samples. The accuracy of their method was 100% 173. Zheng *et al.* used sequential size exclusion chromatography (SSEC) to separate exosomes from human plasma. Matrix-assisted laser desorption/ionization time-of-flight mass spectrometry (MALDI-TOF-MS) data of samples were collected, and a multi-classifier artificial neural network (denoted as Exo-ANN) model was used to identify pancreatic and breast cancer samples. The AUC of their method for PC was 0.86 174. Ko *et al.* developed a multichannel nanofluidic system to isolate exosomes with an ExoTENPO chip, profiled the RNA cargo inside these exosomes, and applied a linear discriminate analysis (LDA) algorithm to identify samples derived from PC patients. Eight exosomal mRNA biomarkers were identified in their mice studies and used to distinguish PC patients from healthy controls. All samples (N=24) in the blinded test set were classified correctly 175.

#### Multi-omics

It is also possible to combine multiple types of biomarkers to detect PC. The analysis of multiple biomarkers revealed the complex molecular landscape of PC and offered the possibility of precision medicine 194-196. Yang *et al.* constructed a multi-analyte panel, including extracellular vesicle miRNAs and mRNAs, cfDNA, and CA19-9. These data are used in the training of various ML algorithms. When applied to PDAC diagnosis, the model achieved an AUC of 0.95 and an accuracy of 92%. Furthermore, the model achieved an accuracy of 84% for disease staging 197. Sinkala *et al.* extracted several types of biomarkers from the TCGA database. Then they used neighborhood component analysis (NCA) to identify biomarker sets. Different biomarkers trained several ML algorithms for PC subtypes differentiation 198. Zhang *et al.* reported a laser desorption/ionization (LDI) mass spectrometry-based liquid biopsy for cancer screening and classification. The study included many cancer types, with 100% accuracy for PC detection in an internal validation cohort 199. Cheng *et al.* deployed ALICE (Automated Liquid Biopsy Cell Enumerator) to identify and enumerate minute amounts of tumor cell phenotypes bestrewed in massive leukocytes and discovered two subpopulations of circulating hybrid cells from PC patients 200. Qiao *et al.* used CT images to train a 2D-3D CNN model for pancreas segmentation and achieved an average DSC of 84.32%. The diagnostic performance (accuracy 87.63%, sensitivity 94.57%, specificity 93.25%, PPV 84.57%, NPV 90.34%) of CT combined with tumor marker (CA-50, CA-199, CA-242) was better than CT or serum tumor markers only 179.

## AI in Prognosis

Accurately predicting the prognosis of PC has important implications for clinical decision-making. This information can help clinicians decide on treatment options, analyze the outcome of pancreatectomy, improve the management of patients, etc. However, classical prognostic factors, such as lymph node status and American Joint Committee on Cancer (AJCC) stage, are not entirely relevant in some long-term survivors 201,202. Also, long-term survivors and general patients did not show significant differences in their mutation profiles 203. These facts make it challenging to predict the prognosis of PC. Due to its excellent computational power, AI was used to analyze PC prognoses, including survival time 204-221, recurrence risk 78,221-224, metastasis 225-230, therapy response 79-81,231-240, etc.

### Survival time

The non-invasive identification of specific imaging features (or signatures) that can predict tumor genomic alterations is termed “radiogenomics,” which integrates radiomics and genomics information. The gene expression profiles obtained in radiogenomics can be used as biomarkers to predict prognosis 241. With the aid of ML, the radiologist used radiological images (CT, MRI) to detect multiple gene expression profiles in PC, including p53 status and PD-L1 expression 204, FAP expression 205, and ITGAV expression 206. These genes had been shown to have predictive ability for the prognosis of PC.

Radiomics can also be applied alone for prognosis prediction. Xu *et al.* used EUS images to predict the prognosis of PC patients undergoing interstitial brachytherapy 207. By extracting the radiomics features of FDG-PET 208,209 or CT 210-212 images and combining them with ML models, researchers could improve the accuracy of survival prediction for PC patients. In addition to direct extraction of image features, CT images have also been used to analyze patient body compositions 213 and tumor heterogeneity 214 in PC to predict survival.

In addition to the radioactive approach mentioned above, some non-imaging methods can predict PC survival. Walczak *et al.* and Aronsson *et al.* combined clinical variables (sex, age, year of diagnosis, tumor stage, etc.) with ANN algorithms to predict survival in PC (91% sensitivity and 38% specificity) 215 and invasive IPMN (82% accuracy and 83% precision) 216, respectively. Using the ML algorithm, Hayward *et al.* combined clinical variables and treatment records to predict PC clinical performance (patient survival time, quality of life scores, surgical outcomes, and tumor characteristics) 217. Biomarker analysis is also a common approach in prognosis. Yokoyama *et al.* evaluated the methylation status of MUC1, MUC2, and MUC4 promoter regions and integrated these results and clinical pathologic features. Then they used SVM-, NN-, and multinomial-based methods to develop a prognostic classifier 218. Winter *et al.* used the NetRank algorithm to filtrate marker genes prognostic for the outcome from PC gene expression profiles. Accuracies were assessed using SVM classifiers and Monte Carlo cross-validation 219. A wavelet-based DL method was proposed by Tang *et al.* to select variables and predict prognosis for PC by training with multi-omics data (genomic, epigenomic, and clinical cohort information). This method predicts prognosis better than the traditional LASSO model (AUC: 0.937 vs. 0.802) 220. Beak *et al.* used multi-omics data to analyze survival and recurrence in PC, with data sources including whole-exome sequencing, RNA sequencing, microRNA sequencing, DNA methylation data, and other clinical data. LR analysis generally revealed the best performance for both disease-free survival (DFS) and overall survival (OS) (accuracy = 0.762 and AUC= 0.795 for DFS; accuracy = 0.776 and AUC = 0.769 for OS) 221.

### Recurrence risk

Clinical features, such as CA19-9 level, tumor location, size, stage, and differentiation degree, are of considerable importance for predicting the risk of recurrence. Li *et al.* collected demographics and various biochemical and pathological variables of PDAC patients from multiple institutions and used six ML algorithms to construct predictive models. SVM and KNN models had the highest accuracy in predicting 1-year and 2-year recurrence (70.9% and 73.4%), respectively 222. Lee *et al.* compared the effects of RF and Cox models on the prognosis of PDAC. Training these two models using multiple clinical variables yielded a mean c-index of 0.6805 and 0.7738 for the RF and Cox models, respectively 78.

In combination with clinical features from patients, radiomics features can be used to predict the risk of recurrence of PC. He *et al.* collected PDAC patients' CT images performed three months after surgery for radiomics analysis. Using clinicoradiological information and radiomics feature jointly or separately, multivariable LR was applied to construct the local recurrences model of PDAC. The combined model achieved an AUC of 0.742 in the validation cohort, which is better than the clinicoradiological-only risk model (AUC 0.533), and the radiomics-only risk model (AUC 0.730) 223. Li *et al.* preprocessed CE-CT and extracted and selected optimal radiomics features from intratumoral volume (ITV) and peritumoral volume (PTV). Then, ANN and LR models were employed to develop the ITV model, PTV model, combined model, clinical model, and radiomics-clinical model. Radiomics-clinical model outperformed other models in predicting 1-year recurrence (AUC 0.764 for validation set) and 2-year recurrence (AUC 0.773 for validation set) 224.

### Metastasis

The lymph node metastasis status of PDAC significantly impacts the choice of treatment options, the risk of postoperative recurrence, and the overall survival rate of patients 242,243. Therefore, correct prediction of lymph node metastasis status can enhance patient prognosis. An *et al.* analyzed preoperative DECT images of regional lymph node dissection in PDAC patients using the Res-Net 18 algorithm to classify lymph node metastasis. The authors compared the prediction effects of virtual monoenergetic images at different voltages. 100 + 150 keV DECT yielded the best predictions (AUC 0.87). If key clinical features (CT-reported T stage, LN status, glutamyl transpeptidase, and glucose) are integrated can further improve the prediction of the model (AUC 0.92) 225. Some studies employed CT radiomics for PC lymph node metastasis prediction, and they all applied multivariable LR to construct a radiomics-based model with AUCs ranging from 0.75 to 0.912 226-228. Shi *et al.* employed T2-weighted (T2WI) and portal venous phase (PVP) MRI images for lymph node metastasis analysis. Radiomics features were extracted by PHIgo software and selected by a gradient-boosting decision tree. T2WI combined with the PVP model has the best performance. The AUCs were 0.834 and 0.807 in the training and validation cohorts, respectively 229.

Liver metastases are more common after PDAC resection but are unpredictable and lead to a poorer prognosis. Zambirinis *et al.* performed liver radiomics analysis on preoperative CE-CT scans and developed an LR classifier to predict early liver metastasis. By incorporating preoperative clinicopathological variables with radiomics features, their model achieved an AUC of 0.76 230.

### Therapy response

AI has also been used to predict treatment responses, including chemotherapy, radiotherapy, immunotherapy, and surgery. Wei *et al.* used a variational autoencoder (VAE) algorithm to extract tumor transcriptome features. Regularized gradient boosted decision trees (XGBoost) were further used to predict chemotherapy drug response for cancer (for PC: AUROC 0.738; AUPRC 0.764) 80. Cos *et al.* collected preoperative activity metrics (step count, heart rate, and sleep time series) from patients with the help of wearable devices and built ML models to predict whether the pancreatectomy achieved the desired outcome (the absence of postoperative pancreatic fistulae, etc.). Their model combined clinical characteristics and achieved an AUC of 0.7875 231. Facciorusso *et al.* developed ANN and LR models to predict pain response after repeat echoendoscopic celiac plexus neurolysis. The predictive performance of the ANN model was higher than the LR model (AUC 0.94 vs. 0.85) 232. Using clinical data and MRI images, Kaissis *et al.* distinguished two subtypes of PDAC (KRT81+ and KRT81-) by gradient boosted-tree algorithm. Subsequently, they assessed chemotherapy response and survival stratified by subtype and radiographic characteristics. Patients with the KRT81+ subtype responded significantly better to gemcitabine-based chemotherapy than FOLFIRINOX (HR 2.33) 81. Schperberg *et al.* combined clinical trials, drug-related biomarkers, and molecular profile information to construct an RF model to predict drug oncologic outcomes in randomized clinical trials. The Spearman correlation (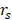) between their predicted model's and actual outcomes was statistically significant (progression-free survival (PFS): 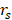 = 0.879, overall survival (OS): 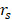 = 0.878, P < .0001) 79. Nasief *et al.* collected CT images of patients during chemotherapy and compared the changes in radiomics features therein. Bayesian-regularization-neural-network was used to build a response prediction model with AUC of 0.98 for kurtosis-coarseness-NESTD (normalized-entropy-to-standard-deviation-difference) combination 233.

Stereotactic body radiotherapy (SBRT) is a therapeutic option in PC care, which permits the precise application of high-dose radiation in 1 to 5 fractions to a limited target volume. It has been proven that SBRT has significantly better outcomes than chemotherapy alone or in combination with conventional external-beam radiotherapy (EBRT) 244. Several studies with radiomics have emerged to predict the response to SBRT. Based on the radiomics features of CT and clinical characteristics, Gregucci *et al.* applied a multivariate LR model to predict local response to SBRT for locally advanced PC (AUC 0.851) 234. Based on CT radiomics features, Parr *et al.* used the gradient boosting machine model to construct OS (c-index 0.66) and recurrence prediction model (AUC 0.78) for PC following SBRT 235. Simpson *et al.* extracted radiomics features from low field strength (0.35 T) MRI for predicting treatment response in PC patients undergoing SBRT. RF algorithm was adopted to construct a prediction model with the AUCs of 0.81 and 0.845 in two similar studies 236,237.

Immunotherapy has shown remarkable efficacy against various tumors, but PC has shown minimal response to immunotherapy. Tumor-infiltrating lymphocytes (TILs) have been proven to be associated with immunotherapy response 245, OS, and PFS 246. Analysis of TILs may help identify PC patients most likely to respond to immunotherapy. Bian *et al.* developed an XGBoost-based model for preoperative prediction TILs in PDAC patients with CT radiomics features (AUC 0.79) 238. Based on MRI radiomics features, Bian *et al.* also predicted Tumor-infiltrating CD8+ T cell 239 and other TILs 240 in PDAC patients with LDA-based model (AUC 0.76) and XGBoost-based model (AUC 0.79), respectively.

Some researchers have used ML to analyze the recovery condition of long-term survivors of PC. Kong *et al.* analyzed CT images by ML to determine changes in body composition (skeletal muscle, adipose tissue) in long-term survivors of pancreatic head cancer. They performed a multi-factor LR analysis of the factors affecting the changes in body composition. Their research concluded that long-term survivors of PC did not recover their preoperative body composition status, and preoperative sarcopenia and recurrence influenced body composition changes 247. The tumor-stroma ratio (TSR) is an independent prognostic factor for solid tumors 248. Based on MRI radiomics features, Meng *et al.* developed and validated an XGBoost classifier for evaluating TSR in patients with PDAC for interstitial targeted therapy selection and monitoring (AUC 0.78 in the validation set) 249.

## Other applications of AI in PC

In addition to the research mentioned above, AI has also been applied to many aspects of PC, including predicting gene mutation 250,251, nucleus segmentation 252, tumor target localization 253, and predicting the risk of ICU admission for PC patients 254, etc.

Electronic health records (EHR) are also considered to be useful for early screening of PC. In a workshop (*Early Detection of Pancreatic Cancer: Opportunities and Challenges in Utilizing Electronic Health Records*) held in March 2021, experts from multiple fields assessed the feasibility of AI-based data extraction and modeling applied to EHRs and identified future directions 255. In another article published the same year, Malhotra *et al.* used logistic regression on EHRs to screen people at high risk of PC. Their method could indicate cancer risk over a decade before diagnosing PC patients 256. Roch *et al.* developed a natural language processing-based pancreatic cyst identification system. It could search and identify keywords of pancreatic cysts based on electronic medical records (EMR), with sensitivity and specificity of 99.9% and 98.8%, respectively. This system could help capture patients at risk of PC 257.

Some studies have also focused on analyzing the omics of PC. Kim *et al.* classified PC using ML and DL to classify quantitative proteomics data 258. Song *et al.* employed AI techniques to deconvolute spatial transcriptomics data to uncover the cell states and subpopulations based on spatial localization 259. Bagante *et al.* integrated whole-exome sequencing data with the help of artificial neural networks for cell-of-origin pattern prediction and molecular subtypes classification of hepato-pancreato-biliary cancers. Combining the clinical data and the above information, they also analyzed the prognosis of cancer patients using random survival forest and Cox analysis 260.

Some studies have attempted to apply DL to estimate medical imaging parameters. Misha *et al.* present an unsupervised physics-informed DL algorithm of intravoxel incoherent motion (IVIM) model called IVIM-NET_optim_ to fit diffusion-weighted imaging (DWI)-MRI data. MRI images of 23 PDAC patients showed IVIM-NET_optim_ superior performance to the least squares and Bayesian approaches at SNRs < 50 60. Ottens *et al.* presented various frameworks, including non-linear least squares (NLLS), Gated Recurrent Unit (GRU), FCN, LSTM, GRU, CNN, and U-Net, that analyze DCE-MRI concentration curves and output extended Tofts-Kety parameter estimates. Testing on 28 PC patients showed that GRU had the best performance 261.

Some researchers have attempted to develop new computer-aided diagnosis (CAD) systems. Li *et al.* constructed a Raman spectroscopic system using CNN models to efficiently distinguish between cancerous and normal pancreatic tissue. The AUCs of all three Raman image-based (1D, 2D Raman images, and 2D Raman PC1) methods were close to 0.99 262. Jadhav *et al.* developed a 3D virtual pancreatography system using ML algorithms for non-invasive diagnosis and classification of pancreatic lesions, the precursors of PC 263. Dmitriev *et al.* developed a CAD system for classifying pancreatic cystic lesions based on RF and CNN. They proposed a visual analytics approach to uncover the system's decision-making process 264. Combining clinical and radiological characteristics, Kang *et al.* built LR and ML models to predict the risk of malignant IPMN. After 200 repetitions, the mean AUCs of their ML and LR models were comparable (0.725 vs. 0.725) 265.

## Challenges and Future Perspectives

**Figure 4:**
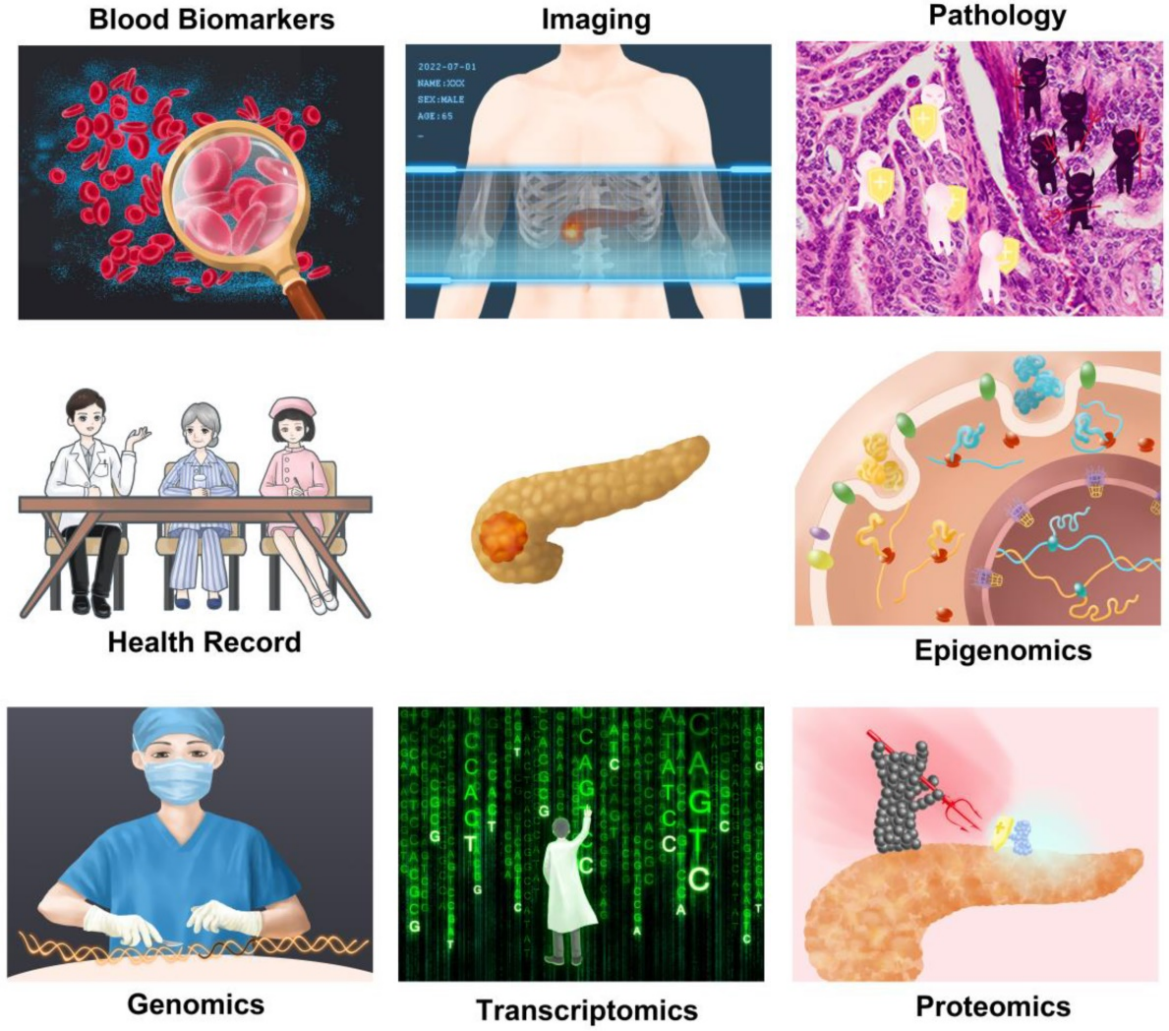
Application of artificial intelligence in multiple related fields of pancreatic cancer. Artificial intelligence can use one type of data alone to make predictions about pancreatic cancer or integrate multi-omics information for analysis.

Although AI has many applications on PC, many challenges have remained (**Figure 4**). Data accessibility and bias may affect the effectiveness of AI predictions. In radiomics, the image's quality may affect the construction of AI models. An assessment of quality gaps in public pancreas imaging datasets found that a substantial proportion of CT images were unsuitable for AI due to biliary stents or other factors 266. Minorities are often underrepresented in clinical trials, leading to biased results 267,268. For AI, insufficient data on minorities may result in the inability to adequately assess patient diversity for algorithm development and testing.

Though radiomics is thought to hold promise for addressing many issues in cancer care, there are still some concerns, such as reproducibility. Variations include intra-individual test-retest repeatability, image-acquisition technique, multi-machine reproducibility, segmentation reproducibility, radiomics feature definition, parameter setting, and implementation. All challenge the reproducibility of radiomics 269-271. To improve the reproducibility of radiomics, scientists have attempted many approaches. The repeatability of radiomics features can be assessed using consistency correlation coefficients (CCCs) 272. In order to build a more reproducible model, only repeatable features should be retained for subsequent model construction. Many studies reuse the dataset from which the model was developed for validation, lacking validation from external datasets. Using an external dataset for validation can improve the reproducibility of the model. Since there are many mature algorithms and software for radiomics, standardizing the process of radiomics, including image acquisition, segmentation, and feature extraction, can help to improve reproducibility 269,271,273.

Due to the specificity of the medicine, an interpretable algorithm is preferred. A flawed algorithm can lead to terrible consequences. Hundreds of hospitals that used IBM Waston Health's cancer AI algorithm for recommending treatments proved to have some errors in their operation 274. However, a trade-off exists between performance and explainability at present. The DL models usually perform better, but they are often the least explainable because they are purely data-driven, and the underlying structures are challenging to interpret 32,275. Three main approaches have attempted to address the interpretability of DL models: (1) proxy models, which use more traditional statistical models to explain the operational properties of DL; (2) visualization, which shows the internal mechanisms of DL models; and (3) internal interpretability approach, where the model can explain by itself which parts are essential 276,277. This suggests that before CAD systems can be used in the clinic, they must be approved for safety and efficacy to avoid patient harm.

As knowledge of the disease continues to expand, the data collected will gradually increase, and clinical decision-making will become more and more complex. Training AI by a single type of medical image or biomarker is not perfect for the diagnosis and prognosis of PC. It should be noted that the study of multimodal features based on image and multi-omics is a new direction for future research. Despite the challenges of AI in PC, it will eventually emerge in all areas of PC due to its great advantage in integrating complex data. In clinical practice, building viable healthcare AI systems requires the joint work of experts in multiple fields, including clinicians, basic scientists, statisticians, and engineers. As AI technology advances and various experts collaborate, its features will become more powerful and accurate.

## Conclusions

Here we summarized the applications of AI on PC. AI-based early screening may be a critical factor in improving the prognosis of PC patients. The combination of medical images and AI may become an essential part of CAD systems to assist physicians in making adequate and accurate diagnoses. In addition, the role of AI in multi-omics and pathology cannot be ignored. With the decrease in computing costs and improved computer technology and biotechnology, AI will make a fantastic process in the medical field. This progress requires a collaborative effort between clinicians, basic scientists, statisticians, and engineers. Despite some limitations, it will still dramatically improve many aspects of PC in the foreseeable future because of its powerful computing capabilities.

## Figures and Tables

**Table 1:** Advantages and disadvantages of imaging modalities for pancreatic cancer

Modality	Advantages	Disadvantages
EUS	High-resolution; Useful in tissue sampling	Invasive; Operator dependent
CT	High spatial resolution; Widely available	Poor contrast resolution
MRI	High contrast resolution; High sensitivity to small tumor and metastasis	Limited availability; Image artifacts
PET	Provides functional metabolic information	Poor spatial and contrast resolution; Physiological FDG uptake disturbance

**Table 2:** Applying AI based on EUS in the differential diagnosis of pancreatic cancer and other pancreatic tumors

Reference	Sample size	Data source	Algorithms	Aim	Best result
Zhu *et al.* 101	388 cases	EUS	SVM	PC vs CP	Accuracy (94.2%), Sensitivity (96.25%), Specificity (93.38%), PPV (92.21%), NPV (96.68%)
Udriştoiu *et al.* 102	65 cases	multi-sequences EUS	CNN, LSTM neural network	PDAC vs CPP vs PNET	AUC (0.98), Accuracy (98.26%)
Tong *et al.* 103	558 cases	CE-EUS	CNN (ResNet-50)	PDAC vs CP	AUC (0.986)
Tonozuka *et al.* 104	1390 images	EUS	CNN	PDAC vs CP	AUC (0.940), Sensitivity (92.4%), Specificity (84.1%), PPV (86.8%), NPV (90.7%)
Sǎftoiu *et al.* 105	167 cases	CEH-EUS	ANN	PC vs CP	Specificity (94.44%), Sensitivity (94.64%), PPV (97.24%), NPV (89.47%)
Marya *et al.* 106	1174461 images from 583 cases	EUS	CNN	AIP vs PDAC vs CP vs NP	**For PDAC:** AUC (0.976), Sensitivity (95%), Specificity (91%), PPV (87%), NPV (97%)
Sǎftoiu *et al.* 107	68 cases	EUS elastography	NN_MLP	PC vs CP vs NP vs PNET	AUC (0.932), Accuracy (89.7%), Sensitivity (91.4%), Specificity (87.9%), PPV (88.9%), NPV (90.6%)
Norton *et al.* 108	35 cases	EUS	NN	PC vs CP	Accuracy (80%), Sensitivity (100%), Specificity (50%)
Sǎftoiu *et al.* 109	258 cases	EUS elastography	ANN_MLP	PC vs CP	AUC (0.94), Accuracy (91.14%), Sensitivity (87.59%), Specificity (82.94%), PPV (96.25%), NPV (57.22%)
Kuwahara *et al.* 110	3,970 images	EUS	CNN (ResNet-50)	Diagnosis of malignancy in IPMN	AUC (0.98), Accuracy (94.0%), Sensitivity (95.7%), Specificity (92.6%), PPV (91.7%), NPV (96.2%)
Iwasa *et al.* 111	100 cases	CE-EUS	U-Net	PC segmentation	Median IoU (0.77)
Zhang *et al.* 112	2207+19486 images	EUS	ResNet	Pancreas segmentation; station recognition	**Pancreas segmentation:** DSC (71.5%); Station recognition: accuracy (82.4%)

**Abbreviations:** AIP: autoimmune pancreatitis; ANN: artificial neural network; AUC: area under the curve; CE-EUS: contrast-enhanced EUS; CEH-EUS: contrast-enhanced harmonic EUS; CNN: convolutional neural network; CP: chronic pancreatitis; CPP: chronic pseudotumoral pancreatitis; DSC: dice similarity coefficient; IPMN: intraductal papillary mucinous neoplasm; IoU: intersection over union; LSTM: long short-term memory; MLP: multilayer perceptron; NN: neural network; NP: normal pancreas; NPV: negative predictive value; PDAC: pancreatic ductal adenocarcinoma; PNET: pancreatic neuroendocrine tumor; PPV: positive predictive value; SVM: support vector machine.

**Table 3:** Application of AI based on CT in the differential diagnosis of pancreatic cancer and other pancreatic tumors

Reference	Sample size	Data source	Algorithms	Aim	Best result
Ma *et al.* 113	3494 images from 190 cases	CE-CT	CNN	PC diagnosis	Accuracy (95.47%), Sensitivity (91.58%), Specificity (98.27%)
Liu *et al.* 114	338 cases	CE-CT	faster R-CNN	PC diagnosis	AUC (0.9632), Precision (76.64%)
Si *et al.* 115	143,945 images from 319 cases	CE-CT	ResNet18, U-net32, ResNet34	PC diagnosis	AUC (0.871), Accuracy (82.7%), Sensitivity (86.8%), Specificity (69.5%)
Qiu *et al.* 116	312 cases	Plain CT	MSTA architecture, SVM	PDAC diagnosis	AUC (0.88), Accuracy (81.19%), Sensitivity (76.64%), Specificity (85.59%)
Qureshi *et al.* 117	216 cases	CE-CT	RFE_NB	PDAC prediction	Accuracy (86%)
Ebrahimian *et al.* 118	103 cases	DECT	RF	Benign vs Malignant Pancreatic Lesions	AUC (0.94), Accuracy (89%), Sensitivity (90%), Specificity (88%)
Chakraborty *et al.* 119	103 cases	CE-CT	RF, SVM	Predict High Risk IPMN	AUC (0.81)
Chu* et al.* 120	380 cases	MDCT	RF	PDAC detection	AUC (0.999), Accuracy (99.2%), Sensitivity (100%), Specificity (98.5%)
Mukherjee *et al.* 121	420 cases	CE-CT	KNN, SVM, RF, and XGBoost	PDAC detection	AUC (0.98), Accuracy (92.2%), Sensitivity (95.5%), Specificity (90.3%)
Polk *et al.* 122	51 cases	CE-CT	LR	IPMN malignancy prediction	AUC (0.93)
Ikeda *et al.* 123	71 cases	CE-CT	NN	PDAC vs mass-forming pancreatitis	AUC (0.916)
Chen *et al.* 124	100 cases	CE-CT	LASSO, SVM (RFE_LinearSVC)	SCN vs MCN	AUC (0.932), Sensitivity (87.5%), Specificity (82.4%)
Ren *et al.* 125	112 cases	CE-CT	RF	PASC vs PDAC	AUC (0.98), Accuracy (94.5%), Sensitivity (98.3%), Specificity (90.1%), PPV (91.9%), NPV (97.8%)
Xie *et al.* 126	216 cases	CE-CT	RF	MCN vs atypical SCN	AUC (0.734), Accuracy (72.8%), Sensitivity (74.8%), Specificity (70.5%), PPV (73.2%), NPV (79.8%)
Li *et al.* 127	97 cases	MDCT	LASSO regression	focal-type AIP vs PDAC	AUC (0.97), Accuracy (94%), Sensitivity (95%), Specificity (93%)
Ziegelmayer *et al.* 128	86 cases	CE-CT	Deep CNN+ Extremely Randomized Trees	AIP vs PDAC	AUC (0.90), Sensitivity (89%), Specificity (83%)
Yang *et al.* 129	78 cases	CE-CT	RF+ LASSO	MCN vs SCN	AUC (0.77), Accuracy (85%), Sensitivity (95%), Specificity (83%)
Gao *et al.* 143	170 cases	CE-CT	mRMR+ LASSO	MCN vs SCN	AUC (0.91), Accuracy (85%), Sensitivity (92%), Specificity (81%)
Panda *et al.* 130	1917 images	Venous phase CT	3D CNN	Pancreas segmentation	DSC (91%), HD (0.15mm)
Mahmoudi *et al.* 131	157 cases	CT	3D CNN, U-Net, TAU-Net	PDAC segmentation	DSC (60.6%), Precision (57.8%), Recall (78.0%), HD (3.73mm)
Huang *et al.* 132	170 cases	CE-CT	U-net	PNET segmentation, grading	DSC (81.8%), AUC (0.87)
Lim *et al.* 133	1006 cases	CE-CT	3D U-Net	Pancreas segmentation and volumetry	DSC (84.2%), Precision (86.9%), Recall (84.2%)
Boers *et al.* 134	1995 images	Venous phase CT	3D U-net	Pancreas segmentation	DSC (78.1%)
Xie *et al.* 135	82 cases	CE-CT	RSTN	Pancreas segmentation	DSC (84.53%)
Wang *et al.* 65	800 images	Venous phase CT	IGA-Net	PDAC segmentation; NP vs PDAC	DSC (60.29%), Sensitivity (99.75%), Specificity (96.50%)
Zhou *et al.* 136	14 cases	4DCT	ResNet-50, FPN	Tumor positioning	DSC (98%)
Abel *et al.* 137	221 images	Venous phase CT	nnU-Net	PCL detection	Sensitivity (78.8%)
Lyu *et al.* 138	47 cases	CE-CT	DLIR-H	PC resectability prediction	AUC (0.91), Sensitivity (97%), Specificity (87%)
Chang *et al.* 139	401 cases	CE-CT	SVM+LASSO	PDAC grading	AUC (0.961), Accuracy (90.1%), Sensitivity (88.6%), Specificity (91.7%), PPV (92.1%), NPV (88.0%)
Luo *et al.* 140	112 cases	CE-CT	CNN	PNET grading	AUC (0.82), Accuracy (82.1%), Sensitivity (88.3%), Specificity (84.6%)
Wan *et al.* 57	137 cases	CT	SAE+ mRMR+ SVM	PNET grading	AUC (0.715, SAE-based model), (0.771, hybrid feature-based model)
Wan *et al.* 58	114 cases	CE-CT	SAE+ SVM/MLR/ANN	PNET grading	AUC (0.845, SVM) (0.856, MLR) (1.00, ANN)

**Abbreviations:** 4DCT: four dimensions CT; AIP: autoimmune pancreatitis; ANN: artificial neural network; AUC: area under the curve; CE-CT: contrast-enhanced CT; CNN: convolutional neural network; CT: computed tomography; DECT: dual energy CT; DLIR: deep learning image reconstruction; DSC: dice similarity coefficient; FPN: feature pyramid network; HD: Hausdorff distance; IGA-Net: Inductive Attention Guidance Network; IPMN: intraductal papillary mucinous neoplasm; KNN: k-nearest neighbor; LASSO: least absolute shrinkage and selection operator; MCN: pancreatic mucinous cystadenoma; MDCT: multidetector CT; MLR: multivariable logistic regression; mRMR: minimum redundancy; MSTA: multiresolution-statistical texture analysis; NB: naïve Bayes; NN: neural network; NP: normal pancreas; PASC: pancreatic adenosquamous carcinoma; PCL: pancreatic cystic lesion; PDAC: pancreatic ductal adenocarcinoma; PNET: pancreatic neuroendocrine tumor; RF: random forest; RFE: recursive feature elimination; RSTN: recursive feature elimination; SAE: sparse autoencoder; SCN: pancreatic serous cystadenoma; SVM: support vector machine.

**Table 4:** AI-based on MRI is applied in the differential diagnosis of pancreatic cancer and other pancreatic tumors

Reference	Sample size	Data source	Algorithms	Aim	Best result
Li *et al.* 146	267 samples from 4 modalities (T1: 67, T2: 68, DWI: 68, AP: 64)	T1, T2, DWI, AP MRI	UDA+ meta learning+ GCN	PC segmentation	DSC (62.08%, T1), (61.35%, T2), (61.88%, DWI), (60.43%, AP)
Chen *et al.* 147	73 cases	multi-sequences MRI	Spiral-ResUNet	PC segmentation	DSC (65.60%), Jaccard index (49.64%), HD (7.27mm), Recall (76.69%), Precision (62.96%)
Liang *et al.* 148	56 DCE MRI sets	DCE MRI	CNN (SGDM)	PDAC segmentation	DSC (71%), HD (7.36mm), MSD (1.78mm)
Goldenberg *et al.* 149	30 mouse models	T1 relaxation, CEST, and DCE MRI	SVM	PC classification	Accuracy (87.5%, CEST) (85.1%, DCE)
Cui *et al.* 150	202 cases	T1-w, T2-w, CET1-w MRI	LASSO	BD-IPMN grading	AUC (0.903), Specificity (94.8%), Sensitivity (73.4%)
Corral *et al.* 151	139 cases	multi-sequences MRI	CNN	IPMN classification	AUC (0.783), Sensitivity (75%), Specificity (78%), PPV (73%), NPV (81%)
Hussein *et al.* 56	171 cases	T2 MRI	SVM, RF, 3D CNN	IPMN classification	Unsupervised:Accuracy (58.04%), Sensitivity (58.61%), Specificity (41.67%); Supervised: Accuracy (84.22%), Sensitivity (97.2%), Specificity (46.5%)
Cheng *et al.* 152	60 cases	CE-CT, T2 MRI	LR, SVM	Malignant IPMN prediction	**MRI+SVM:** AUC (0.940), Accuracy (86.7%), Sensitivity (95.7%), Specificity (81.1%), PPV (75.9%), NPV (96.8%) **CT+SVM:** AUC (0.864), Accuracy (83.3%), Sensitivity (78.3%), Specificity (86.5%), PPV (78.3%), NPV (86.5%)

**Abbreviations:** AUC: area under the curve; BD-IPMN: branching type IPMN; CEST: chemical exchange saturation transfer; CNN: convolutional neural network; DCE: dynamic contrast enhancement; DSC: dice similarity coefficient; GCN: Graph Convolutional Networks; HD: Hausdorff distance; IPMN: intraductal papillary mucinous neoplasm; LR: logistic regression; MLP: multilayer perceptron; MSD: mean surface distance; NPV: negative predictive value; PC: pancreatic cancer; PDAC: pancreatic ductal adenocarcinoma; PPV: positive predictive value; RF: random forest; SGDM: stochastic gradient descent with momentum; SVM: support vector machine; UDA: unsupervised domain adaptation; AP MRI: atrial phase MRI; DWI: diffusion weighted imaging.

**Table 5:** Applying AI based on PET in the differential diagnosis of pancreatic cancer and other pancreatic tumors

Reference	Sample Size	Data Source	Algorithm	Aim	Best result
Li *et al.* 75	80 cases	PET/CT	HFB-SVM-RF	PC diagnosis	Accuracy (96.47%), Sensitivity (95.23%), Specificity (97.51%)
Liu *et al.* 157	112 cases	PET/CT	SVM	PDAC vs AIP	AUC (0.9668), Accuracy (89.91%), Sensitivity (85.31%), Specificity (96.04%)
Zhang *et al.* 158	111 cases	PET/CT	RF, adaptive boosting, SVM	PDAC vs AIP	AUC (0.93), Accuracy (85%), Sensitivity (86%), Specificity (84%)
Xing *et al.* 159	149 cases	PET/CT	XGBoost	PDAC grading	AUC (0.994)

**Abbreviations:** AIP: autoimmune pancreatitis; AUC: area under the curve; CT: computed tomography; NP: normal pancreas; PASC: pancreatic adenosquamous carcinoma; PDAC: pancreatic ductal adenocarcinoma; PET: positron emission tomography; RF: random forest; SVM: support vector machine.

**Table 6:** Application of AI based on pathological examination in the differential diagnosis of pancreatic cancer and other pancreatic tumors

Reference	Sample Size	Data Source	Algorithm	Aim	Best result
Song *et al.* 163	11 images from 7 cases	WSI	DCM+BAYES, KNN, SVM, ANN	SCN vs MCN	Accuracy (90%), Sensitivity (89%), Specificity (91%), PPV (91%), NPV (89%)
Song *et al.* 164	240 images	WSI	SVM	PDAC diagnosis and grading	**For diagnosis:** Accuracy (94.38%), Sensitivity (93.13%), Specificity (95.63%), PPV (95.78%), NPV (93.50%); **For grading:** Accuracy (77.03%), Sensitivity (74.38%), Specificity (79.69%), PPV (79.65%), NPV (75.40%)
Kriegsmann *et al.* 165	201 cases	WSI	CNN	PIN, PDAC identification	Balanced accuracy (73% for non-aggregated; 92% for aggregated)
Niazi *et al.* 166	33 cases	Ki67 stained slides	CNN	PNET identification	Accuracy (96.2%), Sensitivity (97.8%), Specificity (88.8%)
Vance *et al.* 76	31 cases	WSI and MxIF	RF	PDAC cell populations identification	Accuracy (90.0%)
Momeni-Boroujeni *et al.* 167	277images from 75 cases	FNA	MNN	Benign vs malignant pancreatic cytology	Accuracy (100%) **For atypical cases:** Accuracy (77%), Sensitivity (80%), Specificity (75%)
Naito *et al.* 168	532 images	EUS-FNB	CNN	PDAC detection	AUC (0.9836), Accuracy (94.17%), Sensitivity (93.02%), Specificity (97.06%)
Kurita *et al.* 169	85 cases	cyst fluid and EUS-FNA	NN	Benign vs malignant PCLs	AUC (0.966), Accuracy (92.9%), Sensitivity (95.7%), Specificity (91.9%), PPV (81.5%), NPV (98.3%)

Abbreviations: ANN: artificial neural network; AUC: area under the curve; BAYES: Batesian classifier; CNN: convolutional neural network; DCM: direction cumulative map; FNA: fine needle aspiration; FNB: fine needle biopsy; KNN: k-nearest neighbor; MCN: pancreatic mucinous cystadenoma; NPV: negative predictive value; PC: pancreatic cancer; PIN: pancreatic intraepithelial neoplasia; PPV: positive predictive value; MNN: multilayer perceptron neural network; MxIF: cyclic multiplexed-immunofluorescence; NN: neural network; PDAC: pancreatic ductal adenocarcinoma; RF: random forest; PNET: pancreatic neuroendocrine tumor; SCN: pancreatic serous cystadenoma; SVM: support vector machine; WSI: Whole slide imaging.

**Table 7:** Applications of artificial intelligence in biomarker-based pancreatic cancer diagnosis

Reference	Sample Size	Data Source	Algorithm	Aim	Best result
Chen *et al.* 173	28 samples*	DNA-PAINT (exosomes)	LDA	Cancer detection	Accuracy (100%)
Zheng *et al.* 174	220 cases**	MALDI-TOF-MS (exosomes)	ANN	Cancer discrimination	AUC (0.86)
Ko *et al.* 175	28 mice + 34 cases	ExoTENPO chip (exosomes)	LDA	PC diagnosis	Accuracy (100%)
Gao *et al.* 176	199 cases	SELDI-TOF-MS (proteomes)	SVM, KNN, ANN	PC diagnosis	AUC (0.971), Sensitivity (96.67%), Specificity (100%)
Yu *et al.* 177	100 serum samples	SELDI-proteinchip	DT	PC prediction	Sensitivity (88.9%), Specificity (74.1%)
Yang *et al.* 178	913 serum samples	Multiple serum tumor markers	ANN, LR	PC diagnosis	AUC (0.905), Accuracy (83.53%), Sensitivity (90.86%), Specificity (67.50%)
Qiao *et al.* 179	136 cases	CT images+ serum tumor markers	2D-3D CNN	Image segmentation; PC vs CP	**For image segmentation:** DSC (84.32%); **For PC vs CP:** Accuracy (87.63%), Sensitivity (94.57%), Specificity (93.25%), PPV (84.57%), NPV (90.34%)
Cristiano *et al.* 77	34 cases	Cell-free DNA	GBM	Cancer detection	AUC (0.86), Accuracy (67%), Specificity (71%)
Alizadeh Savareh *et al.* 180	671 cases	GEO database (circulating microRNA)	PSO-ANN-NCA	PC diagnosis	Accuracy (93%), Sensitivity (93%), Specificity (92%)
Yu *et al.* 181	501 cases	exLR	SVM	PDAC detection	AUC (0.960), Accuracy (90.43%), Sensitivity (93.39%), Specificity (85.07%)
Almeida *et al.* 182	648 samples	Gene expression microarray	ANN	PDAC prediction	F1-score (0.86), Accuracy (89.66%), Sensitivity (87.6%), Specificity (83.1%)
Yang *et al.* 197	204 cases	Liquid biopsy	KNN, SVM, LDA, LR, and Naive Bayes	PC diagnosis and staging	**For diagnosis:** AUC (0.95), Accuracy (92%), Sensitivity (88%), Specificity (95%); **For staging:** Accuracy (84%), Sensitivity (78%), Specificity (88%)
Sinkala *et al.* 198	185 cases	TCGA database (proteins, mRNAs, miRNAs, and DNA methylation patterns)	NCA, SVM, DT, LR, ET, KNN	PC subtypes differentiation	Accuracy (98.7% for mRNA-based KNN classifier; 97.8% for the DNA methylation-based SVM classifier)
Zhang *et al.* 199	1183 cases***	LDI-MS	SVM	Pan-cancer diagnosis and classification	For PC: Accuracy (100%)

*Including 9 healthy samples, 10 breast cancer samples, 9 PC samples; **Including 79 breast cancer cases, 57 PC cases, 84 healthy controls; ***Including 97 PC cases. **Abbreviations:** ANN: artificial neural network; AUC: area under the curve; CNN: convolutional neural network; CP: chronic pancreatitis; DT: decision tree; DNA-PAINT: DNA points accumulation for imaging in nanoscale topography; ET: ensemble tree; exLR: extracellular vesicles long RNA; GBM: gradient tree boosting; KNN: k-nearest neighbor; LDA: liner discriminate analysis; LDI-MS: laser desorption/ionization mass spectrometry; LR: logistic regression; MALDI-TOF-MS: matrix-assisted laser desorption/ionization time-of-flight MS; MLP: multilayer perceptron; NCA: neighborhood component analysis; PC: pancreatic cancer; PDAC: pancreatic ductal adenocarcinoma; PPV: positive predict value; SELDI-TOF-MS: surface-enhanced laser desorption/ionization time-offlight mass spectrometry; SVM: support vector machine.

## Abbreviations

4DCT: four dimensions CT
AIP: autoimmune pancreatitis
ANN: artificial neural network
AUC: area under the curve
BAYES: Batesian classifier
BD-IPMN: branching type IPMN
CE-CT: contrast-enhanced CT
CE-EUS: contrast-enhanced EUS
CEH-EUS: contrast-enhanced harmonic EUS
CNN: convolutional neural network
CEST: chemical exchange saturation transfer
CP: chronic pancreatitis
CPP: chronic pseudotumoral pancreatitis
CT: computed tomography
DCE: dynamic contrast enhancement
DCM: direction cumulative map
DECT: dual energy CT
DLIR: deep learning image reconstruction
DNA-PAINT: DNA points accumulation for imaging in nanoscale topography
DSC: dice similarity coefficient
DT: decision tree
ET: ensemble tree
exLR: extracellular vesicles long RNA
FNA: fine needle aspiration
FNB: fine needle biopsy
FPN: feature pyramid network
GBM: gradient tree boosting
GCN: Graph Convolutional Networks
HD: Hausdorff distance
IGA-Net: Inductive Attention Guidance Network
IPMN: intraductal papillary mucinous neoplasm
IoU: intersection over union
KNN: k-nearest neighbor
LASSO: least absolute shrinkage and selection operator
LDA: liner discriminate analysis
LDI-MS: laser desorption/ionization mass spectrometry
LR: logistic regression
LSTM: long short-term memory
MALDI-TOF-MS: matrix-assisted laser desorption/ionization time-of-flight MS
MCN: pancreatic mucinous cystadenoma
MDCT: multidetector CT
MLP: multilayer perceptron
MLR: multivariable logistic regression
MNN: multilayer perceptron neural network
mRMR: minimum redundancy
MSD: mean surface distance
MSTA: multiresolution-statistical texture analysis
MxIF: cyclic multiplexed-immunofluorescence
NB: naïve Bayes
NCA: neighborhood component analysis
NN: neural network
NP: normal pancreas
NPV: negative predictive value
PASC: pancreatic adenosquamous carcinoma
PC: pancreatic cancer
PCL: pancreatic cystic lesion
PDAC: pancreatic ductal adenocarcinoma
PET: positron emission tomography
PIN: pancreatic intraepithelial neoplasia
PNET: pancreatic neuroendocrine tumor
PPV: positive predictive value
RF: random forest
RFE: recursive feature elimination
RSTN: recursive feature elimination
SAE: sparse autoencoder
SCN: pancreatic serous cystadenoma
SELDI-TOF-MS: surface-enhanced laser desorption/ionization time-offlight mass spectrometry
SGDM: stochastic gradient descent with momentum
SVM: support vector machine
UDA: unsupervised domain adaptation
WSI: Whole slide imaging
